# Protective effects of ghrelin on kidney tissue in rats with partial ureteral obstruction

**DOI:** 10.3906/sag-1802-17

**Published:** 2019-04-18

**Authors:** Serhan ÇİMEN, Cemal TAŞDEMİR, Nigar VARDI, Burhan ATEŞ, Seda TAŞDEMİR, Ayla ÖZAYDOĞDU ÇİMEN

**Affiliations:** 1 Department of Urology, Malatya Training and Research Hospital, Malatya Turkey; 2 Department of Urology, Turgut Özal Medical Center, İnönü University, Malatya Turkey; 3 Department of Histology, Turgut Özal Medical Center, İnönü University, Malatya Turkey; 4 Department of Chemistry, Faculty of Science, İnönü University, Malatya Turkey; 5 Department of Pharmacology, Turgut Özal Medical Center, İnönü University, Malatya Turkey; 6 Department of Radiology, Malatya Training and Research Hospital, Malatya Turkey

**Keywords:** Partial ureteral obstruction, ghrelin, protective effect, kidney

## Abstract

**Background/aim:**

The aim was to investigate the protective and therapeutic effects of ghrelin, which has antioxidant and antiinflammatory activity, on preventing kidney damage that occurs by induced partial ureteral obstruction in rats.

**Materials and methods:**

Twenty-eight adult male rats were included in the study, and the rats were divided into 4 groups. After the laparotomy operation on the sham group, the ureter was identified in the retroperitoneal area and was duly sutured (n = 7). Ghrelin was administered for seven days intraperitoneally, and after the nephrectomy performed on the 15th day, the rats were sacrificed (n = 7). A partial ureteral obstruction was performed after the laparotomy on the PUO group. The rats were sacrificed after the nephrectomy operation performed on the 15th day (n = 7). A partial ureteral obstruction was formed after the laparotomy followed by seven days of waiting in the PUO + ghrelin group. Ghrelin was given in the dose of 10 ng/kg per day intraperitoneally for the next 7 days, and the rats were sacrificed after the nephrectomy operation performed on the 15th day (n = 7). All groups were evaluated for histological damage and catalase, superoxide dismutase, total glutathione, malondialdehyde, and myeloperoxidase levels were measured in the same tissues.

**Results:**

When the 2nd group and the sham group were compared histologically, it was observed that the damage had increased by a statistically significant level in the partial ureteral obstruction group (P = 0.001). When the group which was ghrelin-treated after the partial ureteral obstruction was compared to the group with just partial ureteral obstruction, the histopathological changes were found to decrease significantly in that group (P = 0.001). While the statistical significance of the levels of CAT, GSH, and MPO enzymes was detected among biochemical changes in the 2nd group when compared to the sham group (P < 0.01), the 3rd group showed a statistically significant difference in the levels of SOD and GSH enzymes compared to the 4th group (P < 0.05).

**Conclusion:**

Ghrelin administration to rats after the formation of an experimental partial unilateral ureteral obstruction reduces tissue damage due to ghrelin’s antiinflammatory and antioxidant effects. Ghrelin administration may prevent tissue damage biochemically and histopathologically in obstructive uropathy cases.

## 1. Introduction 

Urinary tract obstruction is a pathological condition that can occur with the agenesis of the urinary system, the increase in intraluminal pressure, urinary stones, and infection. This can occur in an obstructed ureter, as well as in both ureters, and this may lead to damage to one kidney or both.

The most common cause of unilateral ureteral obstruction in adults is kidney and/or ureteral calculi (1). Upper urinary tract obstructions and treatment spectrum are quite wide pathologies. The level and degree of obstruction and whether it is acute or chronic play an important role in determining the treatment to be planned. Due to the fact that the upper urinary tract obstructions may result in organ loss, they have an important role in urology practice. This obstruction leads to renal parenchymal damage if not eliminated (2). The main purpose of treatment is to maintain the functional reserve of the kidney and/or to provide some recovery by eliminating the obstruction.

Experimental studies aimed at preventive treatments for renal parenchymal damage that may occur following an obstructive uropathy and nephropathy are thought to be more important in the future. Experimental studies created with well-designed partial ureteral obstruction models in rats can show the obstructive uropathy formed also in humans, at a good level (3,4).

Ghrelin was first identified in the stomach of rats by Japanese scientists Kojima et al. in 1999 (5). The ghrelin cells with endocrine functions present in the oxyntic mucosa of the stomach are produced by (X/A) (6,7). This hormone is synthesized, other than the stomach, in the hypothalamus, pituitary, salivary glands, thyroid gland, small intestine, kidneys, heart, central nervous system, lungs, placenta, gonads, immune system, breasts, teeth, and alpha, beta, and epsilon cells of the pancreas (8–12). Ghrelin can be used as a treatment in many diseases such as growth retardation, heart failure, osteoporosis, and immune system suppression (13). Today, ghrelin has been shown to have antiinflammatory and antioxidant effects in light of successful clinical studies (14).

In those studies, it has been shown that, besides these effects, ghrelin has an antioxidant effect, and thus inhibits oxidative stress and apoptosis that can occur in tissues (15,16). Ghrelin has numerous biological effects such as modulating cell proliferation and survival, inhibition of inflammation, regulation of immune functions, and regulation of cardiovascular actions (17–23).

However, in the literature, there is no study investigating the possible protective effects of ghrelin administration in the presence of obstruction for renal damage.

In our study, we aimed to investigate the protective effects of ghrelin in the treatment and/or prevention of the renal damage induced by partial ureteral obstruction (PUO) experimentally formed in rats.

## 2. Materials and methods

Our study was conducted with the approval of the İnönü Experimental and Clinical Research Ethics Committee (Project No: 2007/17). The study was performed in the İnönü University Experimental Animal Research and Application Center, with the contributions of the İnönü University Faculty of Medicine’s Department of Histology and the İnönü University Science Faculty’s Department of Biochemistry. In the study, 28 adult male Wistar albino rats were used, which were approximately 4 months old, fed with standard feed and fountain water, kept in the same room for 12 h in a nighttime environment and for 12 h in a daytime environment, weighing in the range of 250–300 g, and raised in the experimental animal research center.

**Figure 1:**
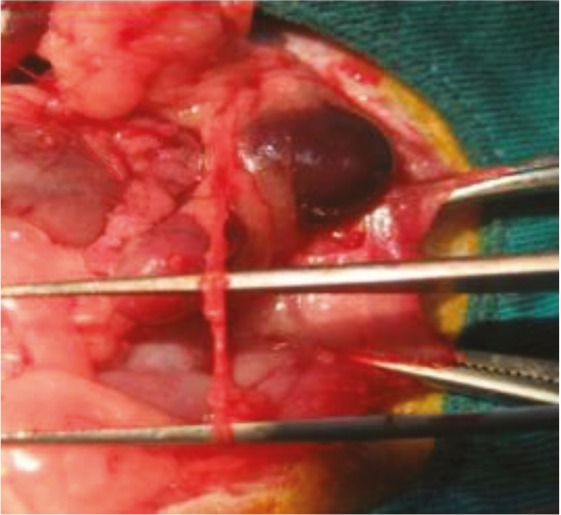
The midline incision in the rats, and the deviation of the intestines in the midline and the presence of the left kidney and ureter.

**Figure 2:**
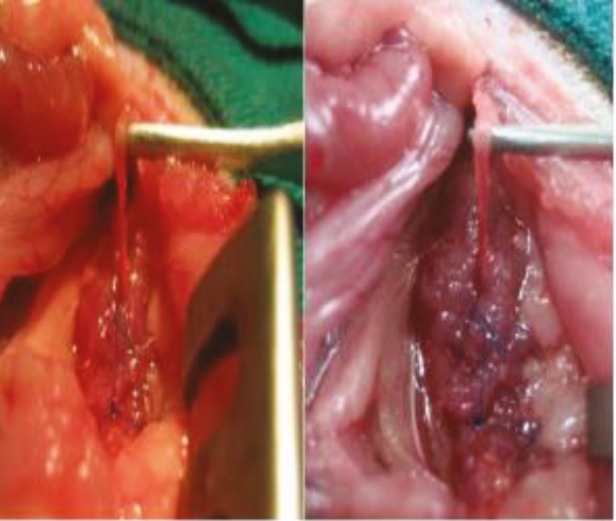
The insertion of the left ureter into the fissure in the psoas muscle and closing of the psoas muscle with the Prolene suture.

All rats were given 100 mg/kg cefazolin sodium intraperitoneally for preoperative surgical prophylaxis. Rats were not fed for 12 h before surgery, but water intake was not limited. In order to create anesthesia, the combination of 40 mg/kg ketamine hydrochloride (Ketalar, Eczacıbaşı, İstanbul) and 8 mg/kg xylazine (Rompun, Bayer AG, Leverkusen, Germany) was administered by intraperitoneal injection before the surgical procedure. PUO was formed according to the technique of Ulm and Miller (24). The abdominal skin of the rats was shaved, and laparotomy was performed with a midline incision under sterile conditions. The intestines were taken medially, and the left kidney and the left ureter were found (Figure 1). After the insertion of the left ureter into the fissure created in the psoas, both sides of the psoas were approached with a 6/0 polypropylene (Prolene, Ethicon) suture (Figure 2). The front wall of the abdomen was closed one by one, with 4/0 silk sutures.

### 2.1. Experimental groups

Each experimental group included 7 rats.

**Sham group**: The rats in this group underwent laparotomy, and the ureters were identified in the retroperitoneal area. They were freed and closed again without any ureter obstruction formation. After the nephrectomy performed on the 15th day, rats were sacrificed (n = 7).

**Ghrelin group**: Partial ureteral obstruction was not formed, but intraperitoneal ghrelin was administered for seven days, and after the nephrectomy was performed on the 15th day, the rats were sacrificed (n = 7).

**PUO group**: The rats in this group underwent laparotomy, and the ureters were found in the retroperitoneal area with partial ureteral obstruction formation. Following this procedure, 7 days were allowed, and 0.5 mL of saline per day was administered intraperitoneally for the next 7 days. After the nephrectomy performed on the 15th day, the rats were sacrificed (n = 7).

**PUO + ghrelin group**: The partial ureteral obstruction was formed, and 7 days were allowed. For the next 7 days, a ghrelin dose of 10 ng/kg per day was administered intraperitoneally, and the rats were sacrificed after nephrectomy performed on the 15th day (n = 7).

At the end of the study, all rats were sacrificed under general anesthesia with high-dose ketamine chloride.

### 2.2. Histological analysis

For histopathological evaluation, the kidney tissues of rats were divided into small sections of 3–4 mm. Hematoxylin and eosin (H&E) stainings were performed in sections to observe the general histological structure. The intertubular hemorrhage, peritubular infiltration, glomerular congestion, and glomerular capillary collapse parameters were evaluated to determine the renal damage. At 20× magnification, 10 sites from each section were examined, and histopathological scoring was determined by the level and the prevalence of renal damage. According to the severity of damage, 0 was evaluated as no change, 1 as mild, 2 as moderate, and 3 as severe.

### 2.3. Biochemical analysis

Catalase (CAT), superoxide dismutase (SOD), myeloperoxidase (MPO), lipid peroxidation (MDA), and total glutathione (tGSH) analyses were performed to determine the enzyme activity in tissues.

### 2.4. Statistical analysis

Statistical analysis was performed with SPSS 17 for Windows. The results were evaluated using one-way ANOVA, Tamhane’s T2, and independent t-tests. Single direction variance analysis was used (one-way ANOVA test), and for multiple comparisons between groups, Tamhane’s and independent t-tests were used. All results were reported as arithmetic mean ± standard error (X ± SE), and P < 0.05 was considered as statistically significant.

## 3. Results

### 3.1. Histopathological findings

**Figure 3:**
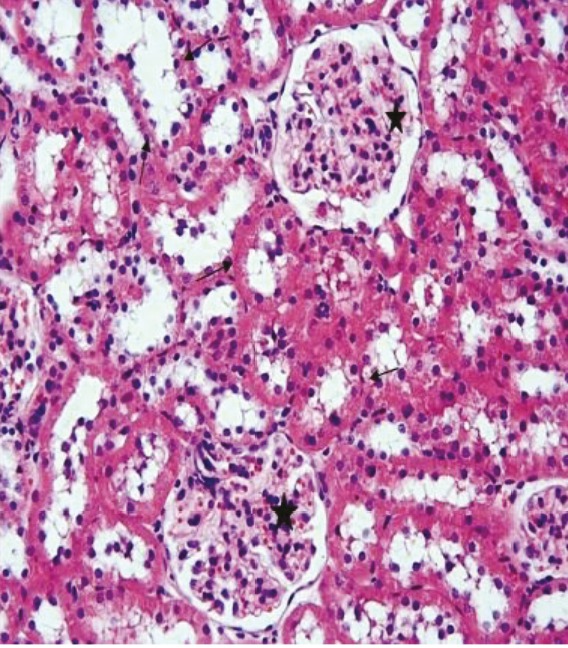
The glomerulus (star) and tubular structures (arrows) in the renal tissue of the control group are observed as normal, H&E, 20×.

**Figure 4:**
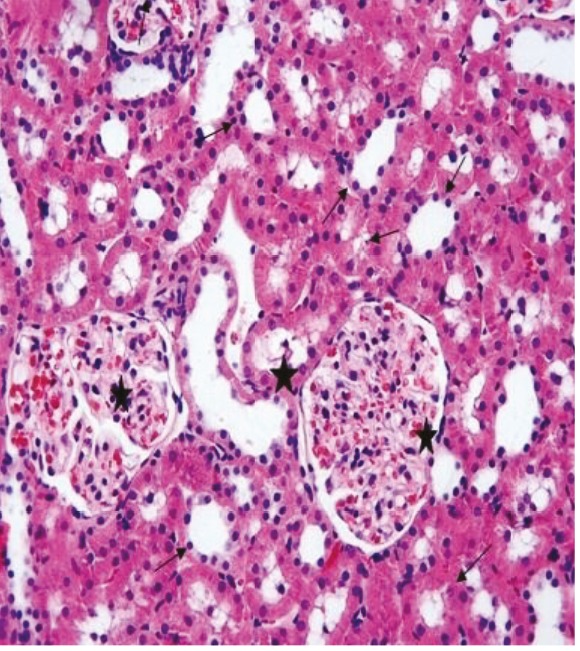
The glomerulus (star) and tubular structures (arrows) in the renal tissue of the ghrelin group are observed as normal, H&E, 20×.

**Figure 5:**
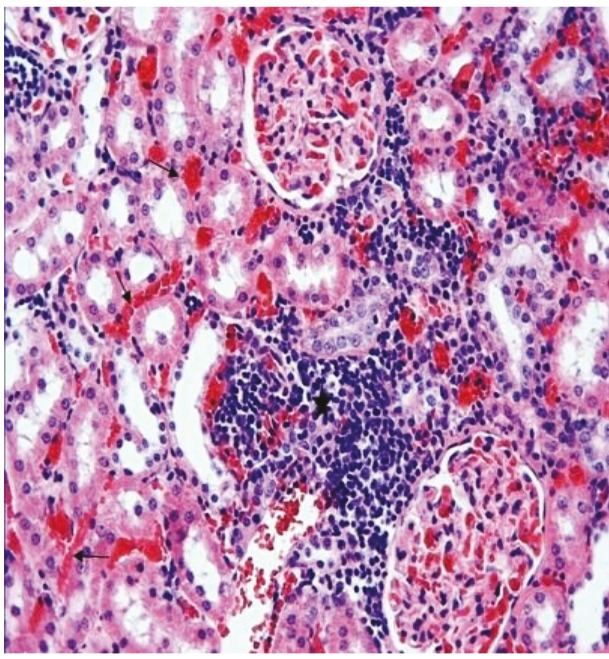
The intertubular hemorrhage (arrows) and infiltration areas (stars) are observed in the renal tissue of the PUO group, H&E, 20×.

**Figure 6:**
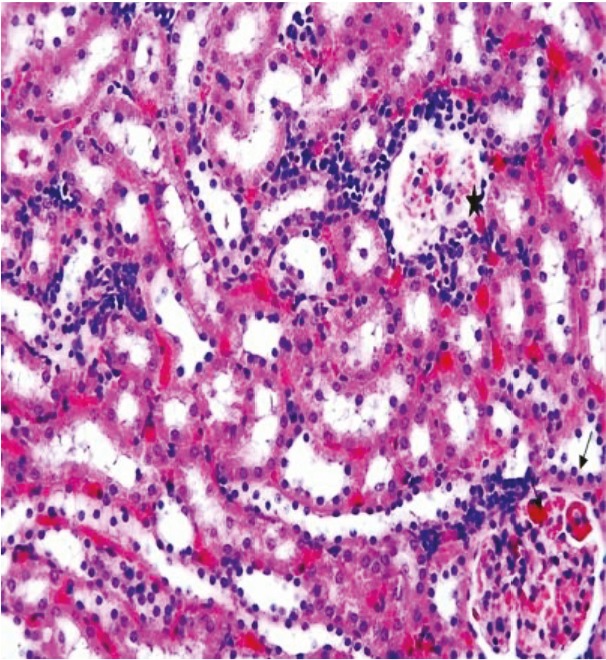
The glomerular congestion (arrows) and collapse (star) are observed in some glomeruli in the PUO group, H&E, 20×.

In the kidney sections of the rats in the sham group, the glomerulus and tubule structures in the cortex were observed to have normal histological structure (Figure 3). In the kidney sections of the rats in the ghrelin group, the histological appearance of the glomeruli and tubules present in the renal cortex was similar to that of the control group (Figure 4). In the kidney tissue samples of the rats in the PUO group, significant hemorrhage and infiltration areas were seen in the intertubular area in the cortex (Figure 5). Changes such as glomerular congestion and collapse were observed in some glomeruli (Figure 6). In this group, the histopathological damage score was calculated as 5.71 ± 0.18. When compared to the control group, it was seen that the damage in this group increased by a statistically significant level (P = 0.001) (Table 1).

**Table 1:** Histological damage scoring of all groups.

Groups	Histological damage
Sham	0.42 ± 0.20
Ghrelin	0.57 ± 0.20
PUO	5.71 ± 0.18a
PUO + ghrelin	1.42 ± 0.52b

a Statistically significant difference with sham group (P = 0.001). b A statistically significant difference with PUO group (P = 0.001).

**Figure 7:**
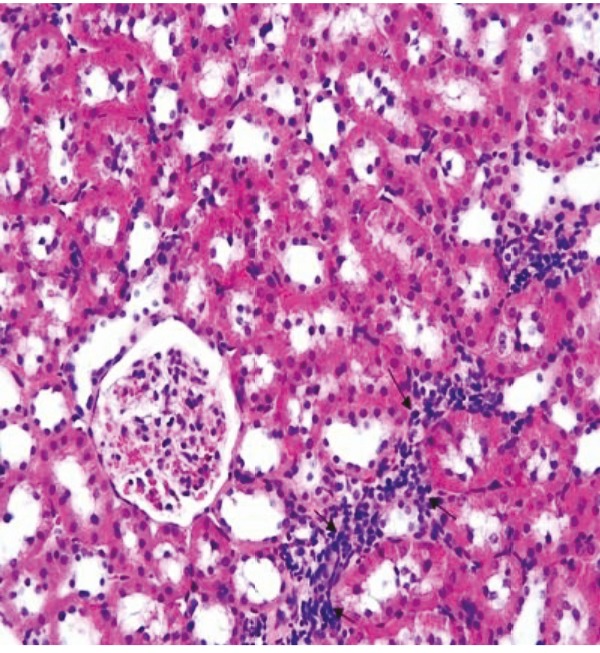
In the PUO + ghrelin group, although renal tissue appears histologically normal, inflammatory inflammation is still observed in some areas (arrows), H&E, 20×.

Although the renal tissue was histologically normal in the renal pathological sections of the rats in the PUO + ghrelin group, local inflammation areas were observed (Figure 7). Compared to the PUO group, the histopathological changes also decreased by a statistically significant level in this group (1.42 ± 0.52) (P = 0.001).

### 3.2. Biochemical findings

When the sham and PUO groups were compared in terms of biochemical findings, a statistically significant change in CAT, GSH, and MPO levels were observed (P < 0.05). However, SOD and MDA levels remained the same. When the PUO and PUO + ghrelin groups were compared, the SOD and GSH levels were found to be statistically significant (P < 0.05). There was no change in CAT, MDA, and MPO levels (Table 2).

**Table 2:** Biochemical analysis results of all groups.

	CAT (U/mg protein)	SOD (U/mg protein)	GSH (nmol/mg protein)	MDA (nmol/g wet tissue)	MPO (U/g wet tissue)
Sham	590.77 ± 56.22	3.82 ± 0.14	3.02 ± 0.28	69.39 ± 19.36	8.65 ± 2.73
Ghrelin	681.77 ± 126.55	4.65 ± 0.37	2.67 ± 0.23	48.53 ± 20.09	8.85 ± 2.41
PUO	484.81 ± 73.32a	3.52 ± 0.39	1.59 ± 0.41a	100.47 ± 22.36	18.54 ± 3.25a
PUO + ghrelin	571.19 ± 126.25	4.16 ± 0.28b	2.77 ± 0.19b	51.90 ± 7.54	10.42 ± 1.62

a Statistically significant difference with sham group (P < 0.05). b A statistically significant difference with PUO group (P < 0.05).

## 4. Discussion

Partial ureteral obstruction is an occlusion that can occur anywhere in the ureter; so, it is a pathology that results in the obstruction of urinary flow (1). If the obstruction is not eliminated, it may progress to kidney loss. Many etiological causes lead to this situation (25). There is an increase in pressure after postrenal obstruction. This increase leads to dilatation in the collecting duct system, increased fibroblast activity in the interstitial area, increase in the mononuclear cells, an increase in the number of macrophages, and an inflammatory process starting in the cells. The formation of free oxygen radicals leads to nephron loss through apoptosis and interstitial fibrosis in the cells, following a chain of events beginning after the release of the cytokines that are not fully elucidated today. These nephrons do not augment because of the lack of mitotic activity after nephron damage occurs in the kidney (26). In the event of being delayed in the obstruction case, permanent damage to the kidney may occur even if the obstruction is eliminated. If not treated, it can cause morbidity and mortality (25). An intervention to any of these stages or any substances going to be given may prevent the progression of nephron damage. If the mechanisms of deterioration in renal function following the elimination of the obstruction can be revealed, the damage in the kidney can be reduced by pharmacological agents to be used for this condition.

There are many studies in the literature performed using antiinflammatory, antioxidant, and antiapoptotic agents to reduce and/or eliminate kidney damage that occurs as a result of PUO.

In a study conducted by Miyajima et al., the effects on renal tubular damage and interstitial fibrosis were examined in rats in which unilateral ureteral obstruction (UUO) was formed by giving etodolac, which is a COX-2 inhibitor, in a dose of 10 mg/kg for 2 weeks. In the obstructed kidney, the tissue prostaglandin E2 (PGE2) production, transforming growth factor beta (TGF-β) level, tubular apoptosis, and interstitial fibrosis were determined to increase. The tissue PGE2 production, TGF-β concentration, tubular apoptosis, and interstitial fibrosis in the group treated with etodolac were observed to decrease significantly. Etodolac has been stated to be a promising agent in preventing UUO-induced renal damage (27).

In another study by Miyajima et al., N-anthranilic acid (tranilast), an antiallergic agent, was initiated on the day before the operation in rats, in which UUO was formed. In the postoperative period, 150 mg/kg was given for 14 days, and tranilast was shown to decrease the tubular apoptosis and increase the tubular proliferation in the tissue, according to the control group. These findings suggest that tranilast is an important agent in terms of preventing the renal tubular damage in UUO (28).

In a study by He et al., UUO was formed in rats, and the effect of losartan on the renal tubular cell apoptosis and renal cell fibrosis was evaluated. As a result, losartan was shown to decrease the renal fibrosis and tubule cell apoptosis in UUO (29). In the study performed by Topçu et al., it was found that verapamil reduced the renal tubular apoptosis in rats in which a partial UUO was formed (30).

In the literature, there are many studies investigating the effects of ghrelin in different experimental models.

In a study performed by Güven et al., ghrelin was given to some rats, which were traumatized and developed acute lung injury. This damage was found to significantly heal. It was concluded that this occurred through the antiinflammatory activity of ghrelin (31). In the neurological injury models created as a result of traumatic brain damage, a significant decrease in apoptosis was observed in ghrelin-treated rats compared to the other groups, and this was thought to be due to the antiinflammatory, antioxidant, and neuroprotective effects of ghrelin (32).

In a study conducted by Golestan et al. for evaluation of the protective role of ghrelin on liver toxicity due to overdose intake of acetaminophen, it was found that ghrelin reduced liver damage by means of the antioxidant and antiinflammatory effects in the given group when compared to other groups (33).

In a study of the protective effects of ghrelin in rats in which paracetamol-induced acute hepatotoxicity was formed, it was observed that in the ghrelin group, the CAT, SOD, and GSH levels increased while the MPO and MDA levels decreased significantly, and histopathological changes showed improvement compared to the control group. It is concluded that these effects were derived from the antioxidant and antiinflammatory effects of ghrelin (34).

In a study conducted by Pamukçu et al., the antiinflammatory activity of ghrelin was analyzed in a rat colitis model formed with dextran sulfate sodium. The MDA level, which is the major indicator of oxidative damage in tissues, and the MPO level, which is the indicator of inflammation, were found to be significantly decreased in the ghrelin group compared to the other groups. In addition, in the ghrelin-treated group, it was determined that the inflammatory cytokines decreased and antiinflammatory cytokines increased in response (35).

In a study conducted by Khowailed et al. about the effects of ghrelin on acute kidney damage caused by sepsis, in the ghrelin group, a decrease in the MPO levels was determined compared to the control group and other groups. Ghrelin was found to treat this damage through its antiinflammatory and antioxidant effects (36).

In another study on the role of ghrelin in rats with acute hepatic toxicity formed with carbon tetrachloride, a significant decrease in tissue MDA and MPO levels was found in the ghrelin group compared to the other groups. On the other hand, it was found to significantly reduce the acute congestion and inflammation in histopathological evaluation (37).

Also, in our study, it was seen that in the rats in which PUO was formed, ghrelin was observed to cause a decrease in kidney glomerular structure congestion, interstitial inflammation, and glomerular hemorrhagic areas histologically compared to the other groups. In the same tissues, there was a decrease in the MPO level, which was involved in the inflammatory process and was released from leukocytes. Moreover, it was observed that the ghrelin treatment decreased the MDA level, which had increased due to the oxidative damage, and increased antioxidant levels such as CAT, SOD, and GSH. It was concluded that ghrelin inhibited these effects through antiinflammatory, antiapoptotic, and antioxidant mechanisms.

In conclusion, despite the use of various agents in the prevention of renal damage after PUO in studies, no standard was established for treatment. This may result from the fact that the cause of kidney damage is not fully established, and studies are still in the experimental stage. In cases where urinary obstruction is encountered clinically, we suggest that ghrelin treatment be used until the obstruction is eliminated so that the obstruction does not cause further loss of function. In order for ghrelin to be used clinically, larger series and multicenter studies are needed.

## Acknowledgment

This study was supported by a grant from the Scientific Research Fund of İnönü University (Project Number: 2013/194).
